# Elevated platelet-to-lymphocyte ratio predicts poor clinical outcomes in non-muscle invasive bladder cancer: a systematic review and meta-analysis

**DOI:** 10.3389/fimmu.2025.1578069

**Published:** 2025-05-13

**Authors:** Wenfeng Hu, Jinze Liang, Jin Luo, Jie Fan, Huaichun Hu, Xinwen Wang, Peng Zhou, Xiaoyi Zhang, Jie Zhou

**Affiliations:** ^1^ The First Clinical Medical School, Hubei University of Chinese Medicine, Wuhan, Hubei, China; ^2^ Department of Urology, Hubei Provincial Hospital of Traditional Chinese Medicine, Affiliated Hospital of Hubei University of Chinese Medicine, Wuhan Hubei, China; ^3^ Hubei Key Laboratory of Theory and Application Research of Liver and Kidney in Traditional Chinese Medicine, Affiliated Hospital of Hubei University of Chinese Medicine, Wuhan, Hubei, China; ^4^ Hubei Provincial Institute of Traditional Chinese Medicine, Wuhan, Hubei, China; ^5^ Department of Orthopedics II, Xingguo County Hospital of Traditional Chinese Medicine, Ganzhou, Jiangxi, China

**Keywords:** platelet-to-lymphocyte ratio, prognosis, biomarker, meta-analysis, non-muscle invasive bladder cancer

## Abstract

**Introduction:**

The prognostic significance of platelet-to-lymphocyte ratio (PLR) in non-muscle invasive bladder cancer (NMIBC) remains controversial despite numerous investigations. This study aimed to systematically evaluate the prognostic value of PLR in NMIBC.

**Materials and methods:**

An extensive systematic search was executed utilizing four major electronic databases: Embase, PubMed, Web of Science, and Cochrane Library. The prognostic significance of PLR was assessed using pooled hazard ratios (HRs) with 95% CIs. Forest plots were used to present data visualization and statistical summaries, illustrating the effects of individual studies and the reliability of the pooled results. Funnel plot analysis and Egger’s test were employed to evaluate the potential presence of publication bias. Sensitivity analysis was performed to assess the robustness of the pooled findings. Subgroup analysis and meta-regression were used to identify sources of heterogeneity.

**Results:**

Eleven retrospective studies encomprising 3,566 patients met the inclusion criteria. Elevated PLR notably correlated with inferior progression-free survival (PFS) (HR=2.132, 95% CI: 1.146-3.967, p=0.017) and relapse-free survival (RFS) (HR=1.732, 95% CI: 1.174-2.554, p=0.006). No statistically meaningful correlation emerged in cancer-specific survival (CSS) (HR=1.218, 95% CI: 0.800-1.854, p=0.358) or overall survival (OS) (HR=1.350, 95% CI: 0.611-2.983, p=0.459). Publication bias was detected in RFS analyses (Egger’s test, P=0.010). Sensitivity analysis demonstrated that the pooled results were robust. Subgroup analysis and meta-regression identified geographic differences and patient characteristics as key sources of heterogeneity in RFS outcomes. Subgroup analysis indicated that geographic differences and sample size were potential sources of heterogeneity in PFS results.

**Discussion:**

This study comprehensively analyzed 11 studies and 3,566 NMIBC cases and found that elevated PLR was significantly associated with poorer RFS and PFS, suggesting that PLR can be used as a prognostic biomarker for the management of NMIBC. The prognostic value of PLR may be related to immune regulation and inflammatory response in the tumor microenvironment; nevertheless, further studies are needed to elucidate its mechanism and establish its clinical application.

**Conclusions:**

This study demonstrates that elevated PLR serves as an independent predictor of poor PFS and RFS in NMIBC patients. As a cost-effective biomarker, PLR shows promise in risk stratification and treatment planning. However, large-scale prospective studies are warranted to validate these findings and establish standardized cut-off values.

**Systematic Review Registration:**

https://www.crd.york.ac.uk/PROSPERO/view/CRD42024621307 **, identifier CRD42024621307.**

## 1 Introduction

In the global cancer landscape of 2023, urinary bladder malignancy emerged as the fourth most common cancer affecting males, representing 6% of new cancer cases and contributing to 4% of cancer-associated mortality worldwide (1). This disease not only elevates the risk of mortality, but also incurs significant healthcare costs and substantially affects patient outcomes and quality of life (2). The clinical classification of bladder cancer follows an invasion-based staging system, distinguishing between muscle-invasive bladder cancer (MIBC) and non-muscle-invasive bladder cancer (NMIBC) (3). NMIBC constitutes 70-75% of all bladder cancer cases (4), characterized by neoplastic involvement limited to either the mucosa layer (Ta stage or carcinoma *in situ*) or submucosa tissue (T1 stage) (3–5). Despite its initial containment, NMIBC demonstrates concerning clinical behavior, with approximately one-fifth of cases advancing to muscle-invasive disease (6), which carries a markedly poorer prognosis (7). Clinical management strategies employ a three-tiered risk classification system - low, intermediate, and high-risk - according to specific clinicopathological criteria (8). For patients classified as intermediate or high-risk, the established treatment protocol combines surgical intervention through transurethral tumor resection with subsequent intravesical immunotherapy using Bacillus Calmette-Guerin (9, 10). Although early detection facilitates surgical management in most cases (70-80%), the disease exhibits concerning patterns of recurrence and progression. Studies indicate that 50-80% of NMIBC cases recur within a five-year window, with 15-30% progressing to advanced stages with poor survival outcomes (11). These statistics highlight the urgent need for reliable, accessible biomarkers to predict disease behavior and improve patient outcomes.

The tumor microenvironment (TME) has received significant attention in cancer research, with a particular focus on its role in tumor development and progression (12). The immune environment is an important component of the TME. The location, density, and phenotype of various immune cells, as well as secreted chemokines and cytokines, comprise the “immune contexture”, which has prognostic significance (13). In addition, inflammatory cell infiltration occurs within the TME, and there is interaction between the immune environment and inflammatory cell infiltration. Across various solid tumors, both inflammatory cell infiltration and the immune environment within the TME have demonstrated substantial prognostic value (14). Among systemic inflammatory markers, the platelet-to-lymphocyte ratio (PLR) has gained substantial recognition in oncological investigations (15). This parameter, derived from the ratio between platelet and lymphocyte counts, serves as a valuable prognostic indicator in multiple cancer types, including non-small cell lung cancer (NSCLC), breast cancer, hepatocellular carcinoma, colorectal cancer, and prostate cancer (16–23).

Nevertheless, the prognostic utility of PLR in NMIBC remains a subject of debate. For example, Caglayan et al. (24) claimed that PLR has limited value in predicting postoperative recurrence in NMIBC patients. Conversely, L. Ding et al. (25) reported that PLR holds prognostic significance in this patient population. In view of these conflicting results, our meta-analysis systematically reviewed 11 cohort studies to quantify the prognostic value of PLR at different levels in NMIBC, using outcome measures such as RFS and PFS. This analysis aims to establish robust evidence for optimizing clinical management strategies and healthcare decision-marking.

## 2 Methods

### 2.1 Study registration

This study was conducted in alignment with the Preferred Reporting Items for Systematic Reviews and Meta-Analyses (PRISMA) guidelines (26). The protocol was pre-registered with the International Prospective Register of Systematic Reviews (PROSPERO) under the identifier CRD42024621307.

### 2.2 Literature search strategy

We performed a systematic search spanning from database inception to November 27, 2024, utilizing Embase, PubMed, Web of Science, and Cochrane Library. Search terms incorporated variations of “non-muscle invasive bladder cancer,” “NMIBC,” “platelet-to-lymphocyte ratio,” and “PLR”. Supplementary searches examined reference lists and grey literature sources. The detailed search strategies is presented in **Supplementary Table S1**.

### 2.3 Inclusion and exclusion criteria

Inclusion criteria: (1) Pathologically confirmed diagnosis of NMIBC; (2) Investigation of PLR as an exposure variable; (3) Provision of sufficient data for hazard ratio (HR) calculation with 95% confidence interval (CI); (4) Publications in English; (5) Observational study design; (6) Assessment of progression-free survival (PFS), recurrence-free survival (RFS), cancer-specific survival (CSS), or overall survival (OS).

Exclusion criteria: (1) Cell experiments, animal studies, case reports, protocols, conference abstracts, editorials, letters, or reviews; (2) Incomplete or erroneous data presentation; (3) Duplicate publications; (4) Inaccessible full-text articles; (5) Overlapping study populations.

### 2.4 Data extraction

Literature management was facilitated through EndNote 21 software. Two independent researchers (Hu and Liang) carried out the initial browse of abstracts and titles according to the predetermined eligibility criteria, followed by full-text reviews of pertinent literature. In cases of selection disagreement, a third researcher (Zhou) provided additional evaluation to reach a final decision. The reviewers independently documented study information using a predefined extraction template, capturing key parameters including publication year, author information, research design, study location, sample characteristics (size, sex ratio, age), PLR cut-off values, patient classification, therapeutic interventions, follow-up duration, survival analysis, and outcome measures.

### 2.5 Quality assessment

Study quality underwent independent evaluation by two investigators using the Newcastle-Ottawa Scale (NOS) (27), examining three domains: selection, comparability, and exposure. Studies scoring ≥6 points (maximum 9) were designated as high-quality investigations.

### 2.6 Statistical analysis

STATA 15.1 software was used for statistical analysis. The correlation of PLR to RFS, PFS, OS, and CSS was assessed by calculating HR and 95% CI. Heterogeneity assessment combined I^2^ statistics and Cochrane’s Q test (significant: I^2^>50% or P<0.10). The choice between random-effects and fixed-effects models was contingent upon heterogeneity assessment, with the former employed in cases of significant heterogeneity. Robustness of findings was evaluated through sensitivity analyses. Heterogeneity sources were examined via predefined subgroup analyses (stratified by region, sample size, patient characteristics, immunotherapy status, NOS score, PLR cut-off value, and cut-off determination) and meta-regression. Publication bias evaluation combined visual funnel plot assessment with Egger’s test quantification (significance: P<0.05).

## 3 Results

### 3.1 Literature search outcomes

**Figure 1:**
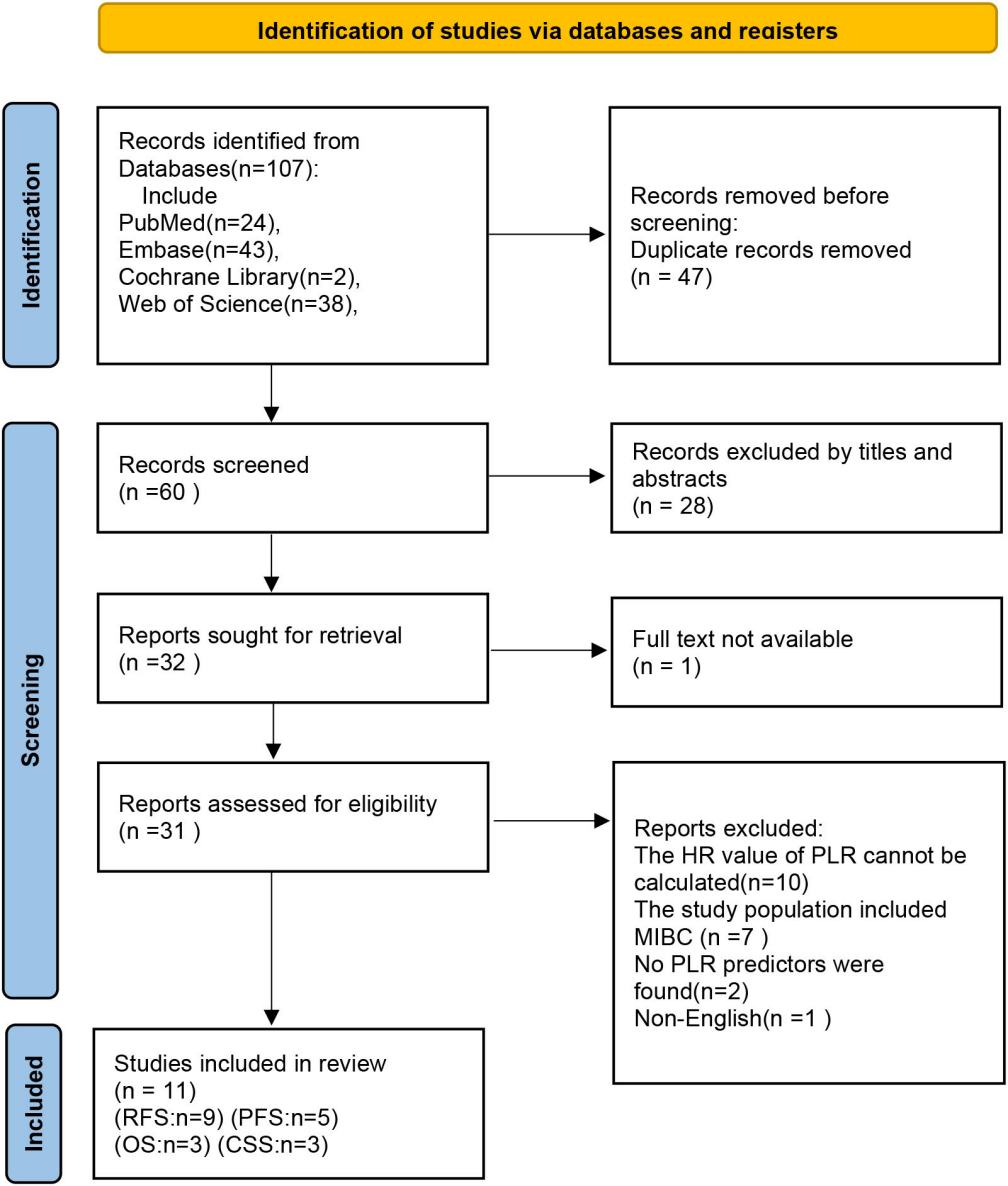
Flow chart of the screening process.

Initial database searches identified 107 publications. Upon removing 47 duplicates and screening titles and abstracts, 32 articles qualified for full-text evaluation. Based on eligibility criteria, 32 articles were further excluded (**Supplementary Table S2**). Finally, 11 studies were included in the meta-analysis. The complete selection workflow is documented in **Figure 1**.

### 3.2 Studies characteristics and quality assessment

The analysis incorporated 11 retrospective cohort studies (24, 25, 28–36) conducted across four countries (China, America, Turkey, and Korea), encompassing 3,566 patients (2,980 males, 586 females). The studies, published between 2017 and 2024, employed PLR cut-off values ranging from 93.075 to 175.71. The quality assessment revealed that NOS scores for all included studies exceeded 6 (**Supplementary Table S3**), confirming their high methodological quality. Detailed study characteristics are summarized in **Table 1**.

**Table 1:** The basic characteristics of the included literature.

Author	Publication year	Study Design	Country	Sample size	Sex (M/F)	Age (year)(M ± SD)	Tumor grade N %)	Tumor number N %)	Tumor size N %)	Type of patient	Treatment method	Follow-up (months)(M ± SD)	Cut-off value (PLR)	Cut-off determination	Outcome index	Survival analysis	NOS score
A. Çaglayan (24)	2023	retrospective cohort	Turkey	74	64/10	67.41 ± 11.31	Low 48(64.9): High 26(35.1)	NA	NA	high-risk NMIBC	TURBT + intravesica chemotherapy + immunotherapy	38.77 ± 19.53	123	ROC curve analysis	OS CSS RFS PFS	Multivariate	8
M. A. Chakra (28)	2024	retrospective Cohort	America	115	96/19	73.4 ± 10.5	NA	NA	NA	high-risk NMIBC	TURBT + BCG + sequential intravesical gemcitabine/docetaxel	23.15 ± 4.51	NA	NA	RFS	Univariate	7
L. Ding (25)	2023	retrospective cohort	China	292	245/47	66 ± 11.92	Low 124(42.466) High 168(57.534)	Single 134(45.890): Multiple 158(54.110)	NA	NMIBC	TURBT + postoperative intravesical chemotherapy	NA	175.71	X-tile program	RFS PFS	Multivariate	7
M. Kang (29)	2017	retrospective cohort	Korea	1551	1302/249	64.65 ± 11.13	PUNLMP 52(3.4): Low 738 (47.8); High 755(48.9): Missing 6	Single 832 (53.6): Multiple 152(46.3)	<3cm 1283(82.9): 3cm 265(17.1): Missing 3	NMIBC	TURBT	52.04 ± 8.21	124	NA	OS CSS	Multivariate	8
S. Y. Mao (30)	2017	retrospective cohort	China	207	169/38	68.46 ± 15.68	G1+G2 130(63): 3 77(37)	Single 129 (62): Multiple 78(38)	<3cm 157(76): 3cm 50(24)	NMIBC	TURBT + intravesica chemotherapy	21.14 ± 3.64	123	ROC curve analysis	RFS PFS	Multivariate	8
C. Wang (31)	2023	retrospective cohort	China	222	180/42	NA	Low 143(64.4): High 79(35.6)	≤ 3 192 (86.5): >3 30(13.5)	≤3cm 171(77.0): >3cm 51(23.0)	NMIBC	TURBT + intravesica chemotherapy	41.38 ± 5.60	93.075	ROC curve analysis	RFS	Multivariate	8
X. Y. Wang (32)	2024	retrospective cohort	China	99	88/11	68.94 ± 11.64	Low 49(49.5): High 50(50.5)	<3 66(66.7): ≥3 33(33.3)	<3cm 64(64.6): ≥3cm 35(35.4)	NMIBC	TURBT + intravesical instillation of gemcitabine	27.71 ± 10.76	157.965	ROC curve analysis	RFS	Multivariate	8
R. Wu (33)	2022	retrospective cohort	China	197	170/27	64.17 ± 11.05	Low 55 (27.9): High 142 (72.1)	Single 86 (43.7): Multiple 111 (56.3)	≤3cm 110(55.8): >3cm 87(44.2)	NMIBC	TURBT+BCG	24.46 ± 2.75	99.8(RFS)/112.7(PFS)	ROC curve analysis	RFS PFS	Multivariate	8
X. Yi (34)	2023	retrospective cohort	China	348	274/74	68.41 ± 11.50	PUNLMP 51 (14.7): UCC-LG 179 (51.4): UCC-HG 118 (33.9)	Single 190 (54.6): ultiple 158 (45.4)	<3cm 207(59.5): 3cm 141(40.5)	NMIBC	TURBT + intravesica chemotherapy	55.43 ± 20.95	150	ROC curve analysis	RFS	Multivariate	8
I. Ö. Yilmaz (36)	2024	retrospective cohort	Turkey	76	65/11	66.28 ± 9.58	G3	Single 34 (44.74): ultiple 42(55.26)	<3cm 43(56.58): 3cm 33(43.42)	NMIBC	TURBT	43.89 ± 31.54	150	reference publications	RFS	Multivariate	8
H. D. Yuk (35)	2019	retrospective cohort	Korea	385	327/58	72.6 ± 10.6	None 9(2.3): Low 50(13.0): High 326(84.7)	Single 148 (38.4): ultiple 237(61.6)	<3cm 290(75.3): 3cm 95(24.7)	high-risk NMIBC	TURBT+BCG	80.00 ± 50.70	171	ROC curve analysis	OS CSS	Multivariate	8

NMIBC, non-muscle invasive bladder cancer; PLR, platelet-to-lymphocyte ratio; RFS, relapse-free survival; CSS, cancer-specific survival; OS, overall survival; NOS, Newcastle-Ottawa Scale; NA, not applicable.

### 3.3 Prognostic significance of PLR for RFS

**Figure 2:**
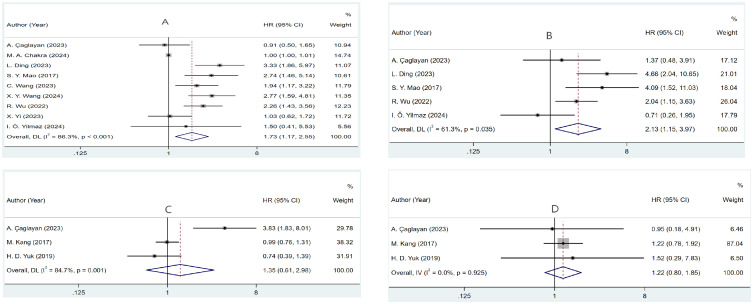
Forest plots of the prognostic role of PLR for **(A)** RFS, **(B)** PFS, **(C)** OS, and **(D)** CSS in NMIBC patients.

Nine studies (24, 25, 28, 30–34, 36) involving 1,630 patients investigated the relationship between RFS and PLR in NMIBC patients. Meta-analysis using a random-effects model (necessitated by substantial heterogeneity: I^2^ = 86.3%, P <0.001) demonstrated significantly inferior RFS in patients with elevated PLR (HR=1.732, 95% CI: 1.174-2.554, p=0.006). Subsequent subgroup analyses and meta-regression identified geographical variation and patient characteristics as potential sources of heterogeneity. These findings are visualized in **Figure 2A** and detailed in **Table 2**.

**Table 2:** Subgroup analysis of prognostic role of PLR for RFS in NMIBC patients.

Subgroups	No. of studies	No. of patients	HR (95% CI)	P	Heterogeneity		Meta-regression p
					I^2^(%)	P-value	
Total	9	1630	1.732(1.174, 2.554)	0.006	86.3	<0.001	
Region							0.018
Europe and America	3	265	1.000(0.995, 1.005)	1.000	0.0	0.789	
East Asia	6	1365	2.165(1.551, 3.022)	<0.001	56.9	0.041	
Sample size							0.580*
<200	5	561	1.512(0.910, 2.514)	0.111	84.4	<0.001	
≥ 200	4	1069	2.030(1.214, 3.394)	0.007	70.8	0.016	
Type of patient							0.018
high-risk NMIBC	2	189	1.000(0.995, 1.005)	0.998	0.0	0.757	
NMIBC	7	1441	2.125(1.556, 2.902)	<0.001	49.4	0.065	
with or without Immunotherapy							0.161
Yes	3	386	1.260(0.743, 2.136)	0.391	83.6	0.002	
No	6	1244	2.099(1.426, 3.089)	<0.001	57.4	0.038	
NOS score							0.901*
≤7	2	407	1.759(0.542, 5.705)	0.347	93.9	<0.001	
>7	7	1223	1.758(1.241, 2.491)	0.002	59.0	0.023	
Total	8	1515	1.908(1.359, 2.680)	<0.001	62.3	0.01	
Cut-off value							0.689*
<150	4	700	1.832(1.188, 2.826)	0.006	60.8	0.054	
≥150	4	815	1.998(1.069, 3.735)	0.030	72.3	0.013	
Cut-off determination							0.405
Others	2	368	2.794(1.465, 5.330)	0.002	16.1	0.275	
ROC curve	6	1147	1.774(1.219, 2.582)	0.003	65.7	0.012	

CI, confidence interval; HR, hazard ratio; NOS, Newcastle-Ottawa Scale; NMIBC, non-muscle invasive bladder cancer; PLR, platelet-to-lymphocyte ratio; RFS, relapse-free survival.

*Represents continuous variable meta regression P value.

### 3.4 Prognostic significance of PLR for PFS

Analysis of five studies (24, 25, 30, 33, 36) revealed that increased PLR values were associated with reduced PFS outcomes (HR=2.132, 95% CI: 1.146-3.967, p=0.017). Notable between-study variance (I^2^ = 61.3%, p=0.035) warranted random-effects modeling. Further investigation through subgroup analyses identified geographical variation and sample size as key sources of heterogeneity (**Figure 2B**, **Table 3**).

**Table 3:** Subgroup analysis of prognostic role of PLR for PFS in NMIBC patients.

Subgroups	No. of studies	No. of patients	HR (95% CI)	P	Heterogeneity	
					I^2^(%)	P-value
Total	5	846	2.132 (1.146,3.967)	0.017	61.3	0.035
Region						
Europe and America	2	150	0.975 (0.471,2.016)	0.945	0.0	0.378
East Asia	3	696	2.132 (1.146,3.967)	<0.001	36.6	0.206
Sample size						
<200	3	347	1.404 (0.754,2.614)	0.284	38.2	0.198
≥200	2	499	4.417 (2.341,8.335)	<0.001	0.0	0.843
with or without Immunotherapy						
Yes	2	271	1.864 (1.127,3.083)	0.015	0.0	0.512
No	3	575	2.434 (0.767,7.724)	0.131	77.9	0.011
Cut-off value						
<150	3	478	2.206 (1.330,3.657)	0.002	14.7	0.309
≥150	2	368	2.132 (1.146,3.967)	0.507	87.5	0.005
Cut-off determination						
Others	2	368	2.132 (1.146,3.967)	0.507	87.5	0.005
ROC curve	3	478	2.206 (1.330,3.657)	0.002	14.7	0.309

CI, confidence interval; HR, hazard ratio; NMIBC, non-muscle invasive bladder cancer; PLR, platelet-to-lymphocyte ratio; RFS, relapse-free survival.

### 3.5 Prognostic significance of PLR for OS

Three studies (24, 29, 35) examined PLR’s relationship with OS. Statistical analysis utilizing random-effects modeling, prompted by marked heterogeneity (I²=84.7%, P=0.001), found no definitive link between PLR and OS (HR=1.350, 95% CI: 0.611–2.983, p=0.459) (**Figure 2C**).

### 3.6 Prognostic significance of PLR for CSS

Examination of CSS across three studies (24, 29, 35) showed minimal statistical heterogeneity (I^2^ = 0.0%, P=0.925), supporting fixed-effects analysis. The data indicated no substantial relationship between PLR and CSS (HR=1.218, 95% CI: 0.800–1.854, p=0.358) (**Figure 2D**).

### 3.7 Sensitivity analysis

**Figure 3:**
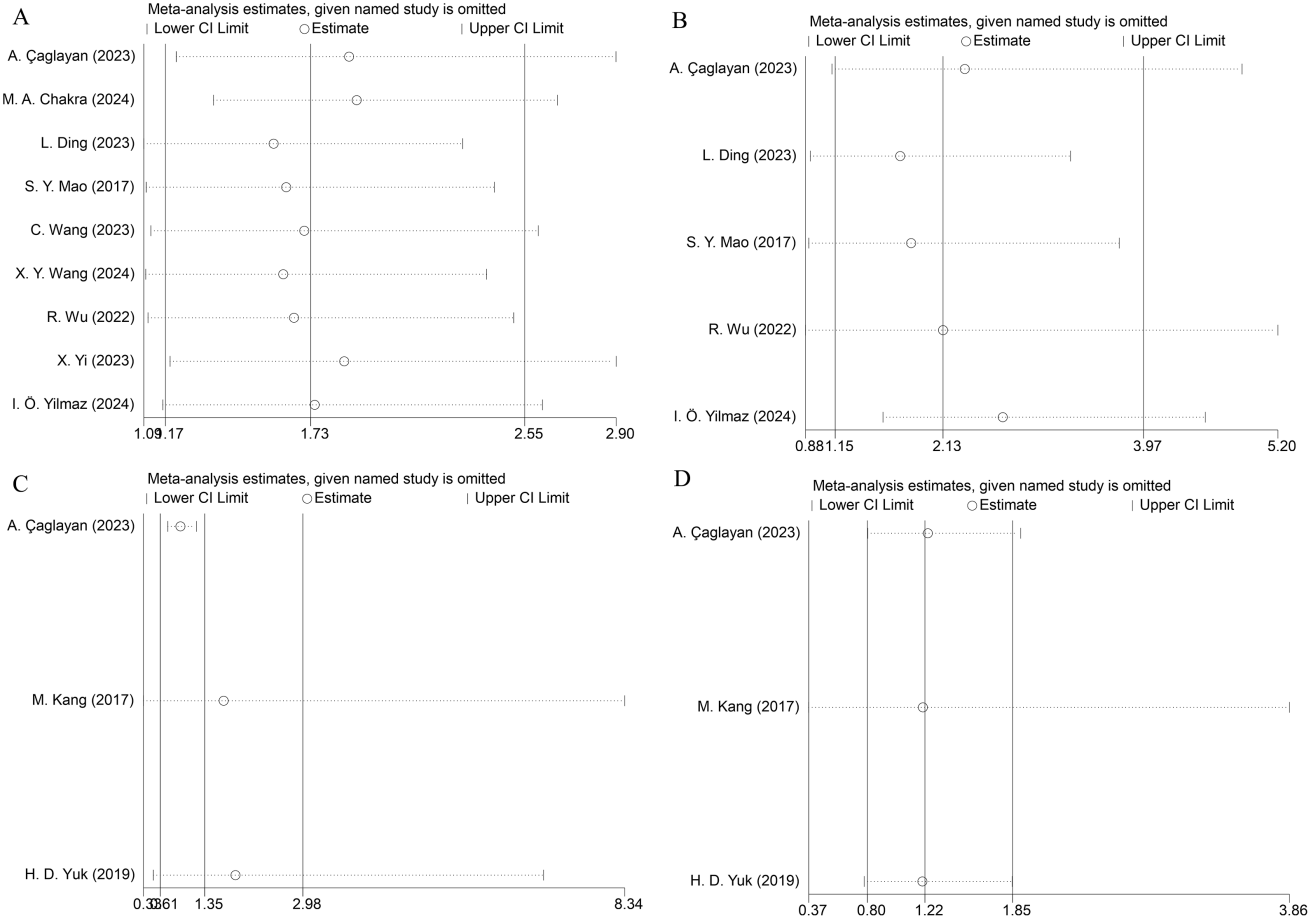
Results for sensitivity analysis. **(A)** RFS, **(B)** PFS, **(C)** OS, and **(D)** CSS.

To verify result stability, we executed leave-one-out sensitivity analyses. Sequential omission of individual studies demonstrated no substantial alterations in the pooled effect estimates, confirming the robustness of our findings (**Figure 3**).

### 3.8 Publication bias

**Figure 4:**
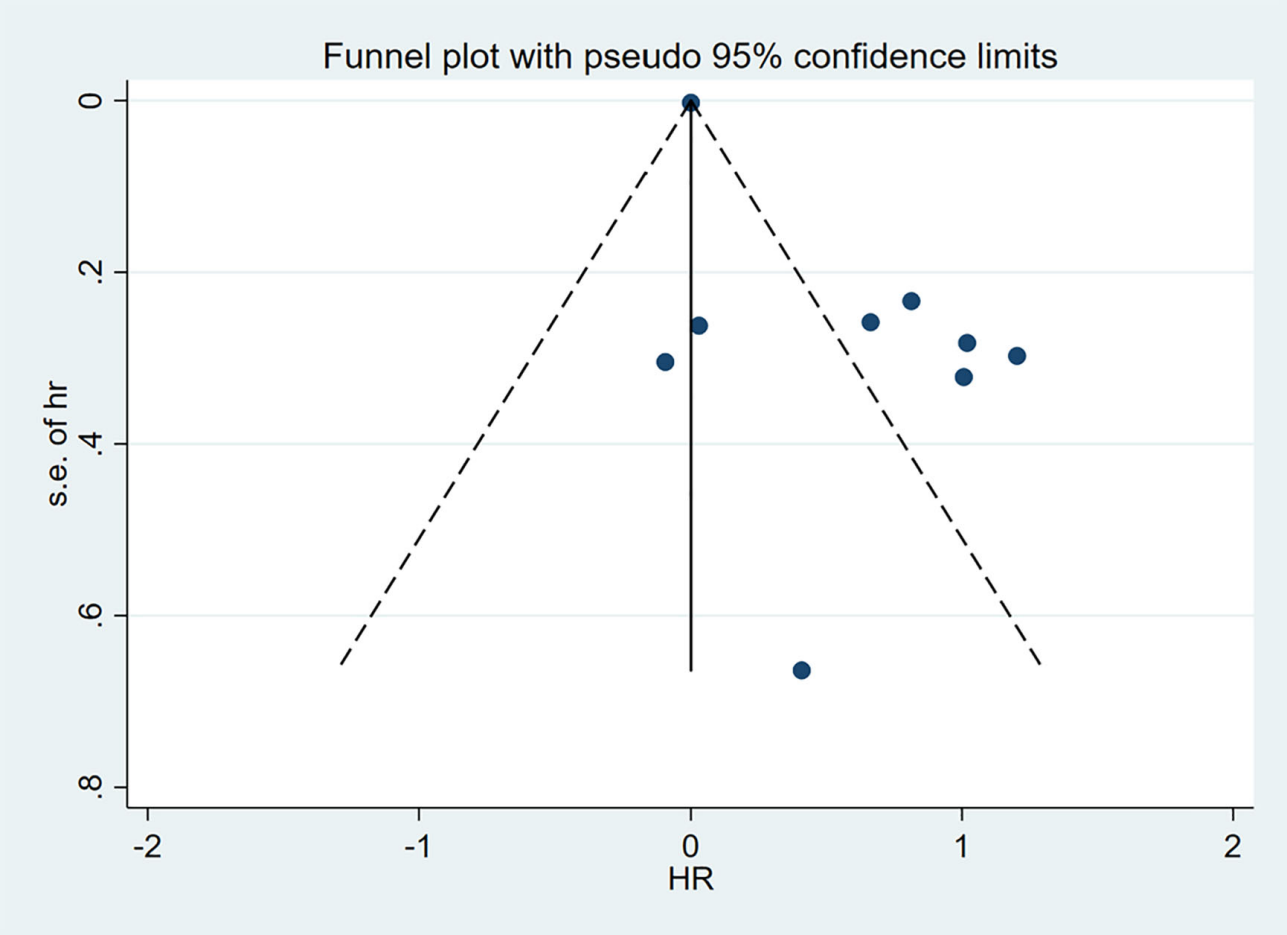
Funnel plot for RFS.

**Figure 5:**
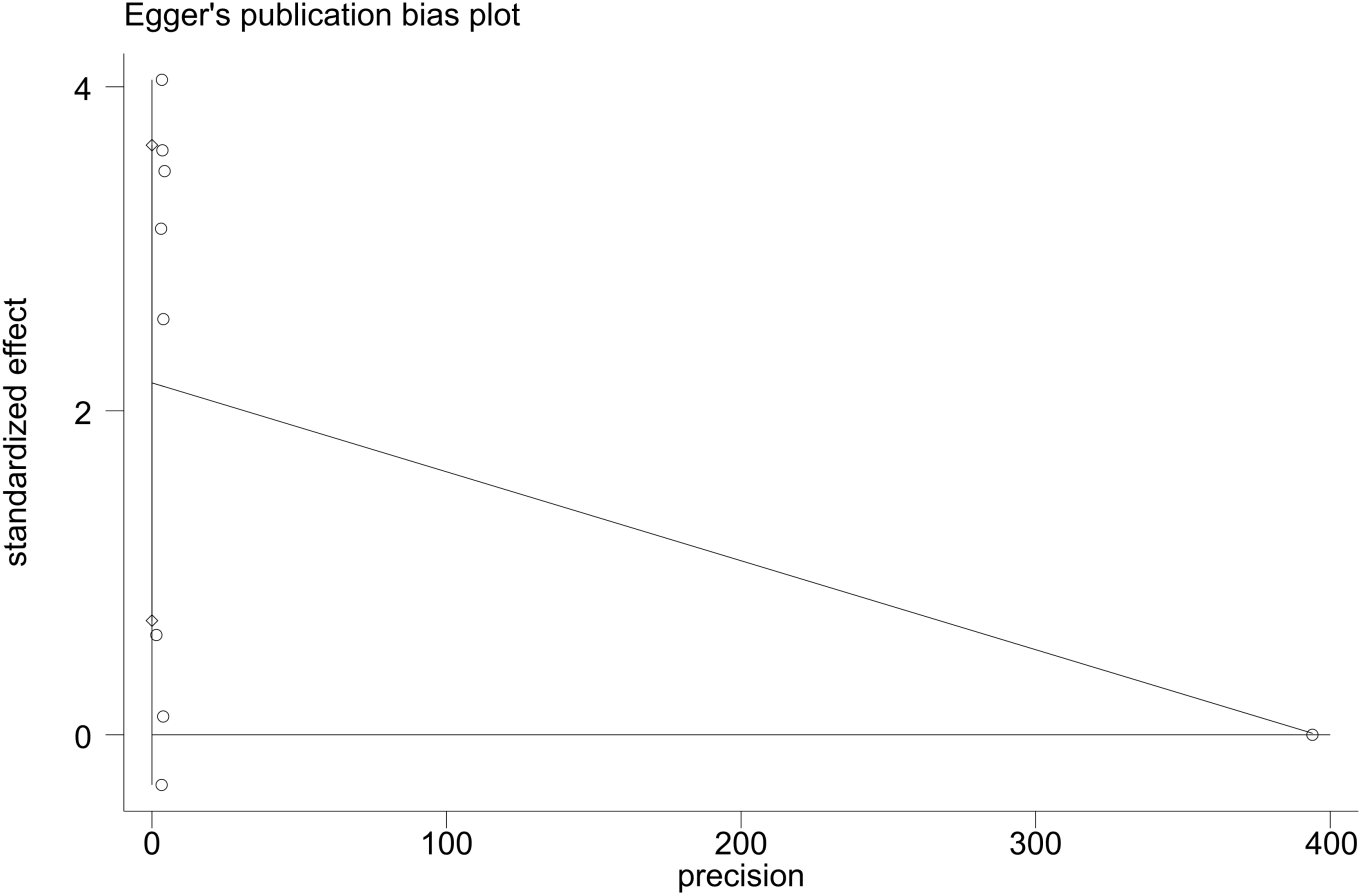
Egger's test for RFS.

Visual examination of funnel plot demonstrated asymmetrical distribution, and Egger’s test (p=0.010) suggested possible reporting bias within the analyzed literature (**Figures 4**, **5**).

## 4 Discussion

This systematic analysis consolidated evidence from 11 studies, comprising 3,566 NMIBC cases, to establish PLR’s prognostic implications in NMIBC. Sensitivity analysis demonstrated that the results of pooling these 11 independent studies were robust, thereby enhancing the reliability of our conclusions. Our findings revealed that elevated PLR correlates significantly with inferior RFS and PFS outcomes, suggesting its potential utility as a readily accessible and reliable prognostic biomarker in NMIBC management.

The prognostic value of PLR has been well-documented across various cancers (37, 38). Recent investigations have demonstrated significant associations between elevated PLR and adverse outcomes in multiple cancer types. For instance, elevated PLR levels correlate with decreased RFS in ovarian cancer (P=0.018) (39) and biliary tract cancer (HR=1.53, 95% CI: 1.16-2.00, p=0.002) (40). Similarly, meta-analyses have shown that higher PLR is associated with shorter PFS in both immunotherapy-treated gastric cancer patients (HR=1.52, 95% CI: 1.20-1.94, P<0.01) (41) and NSCLC patients undergoing immune checkpoint inhibitor therapy (HR=1.57, 95% CI: 1.30-1.90, P < 0.001) (42). The results of our study regarding the prognostic relevance of PLR in NMIBC are consistent with these earlier findings.

The biological basis for PLR’s prognostic value likely stems from the inflammatory nature and immune regulation of the TME, which critically influences tumor initiation, progression, and metastasis (43, 44). The intricate relationship between inflammatory responses and tumor progression has been well-established (45), though the precise mechanisms underlying PLR’s impact on NMIBC survival remain incompletely understood. Platelets contribute to tumor progression through multiple pathways, including the secretion of pro-angiogenic factors (vascular endothelial growth factor, platelet-derived growth factor) (46–48) and the release of microvesicles that activate mitogen-activated protein kinases signaling (49). Research has shown that nucleotides released by platelets promote cancer cell migration across endothelial barriers and enhance metastatic spread through P2Y2 receptor-mediated mechanisms (50). Furthermore, inflammation initiates the recruitment and activation of numerous immune cells, which produce and release a variety of inflammatory mediators, generating an autonomous loop of chronic inflammatory responses. As chronic inflammation progresses, an immunosuppressive microenvironment is induced, characterized by the accumulation of immune suppressor cells, such as MDSCs, pro-inflammatory cytokines, and growth and angiogenic factors that suppress T-cell and natural killer (NK) cells activity (51). Platelet-derived transforming growth factor-beta down-regulates NKG2D, thereby inhibiting NK cell antitumor reactivity (52). Platelets and fibrin (ogen) further increase metastatic potential by impeding NK cell-mediated elimination of tumor cells (53). Conversely, lymphocytes execute crucial anti-tumor functions through suppression of tumor cell division and direct cytotoxic effects (54, 55). NK cells and cytotoxic T lymphocytes (CD8+ T cells) contribute to the antitumor response (13). NK cells recognize tumor cell surface stress molecules (such as MICA/B) via NKG2D receptors, releasing perforin and granzyme to mediate tumor inhibition. CD8+ T cells specifically recognize the antigenic peptide-MHC-I complex through the T cell receptor (TCR), triggering TCR signaling and inhibiting tumor cells; CD8+ T cells can also directly induce tumor cells apoptosis through two main pathways: perforin-granase pathway and death receptor pathway. In addition, they secrete IFN-γ and TNF-α, which indirectly enhance antitumor immunity. Clinical evidence demonstrates that higher levels of lymphocyte infiltration within tumor sites correlate with better treatment response, while reduced lymphocyte presence diminishes anti-tumor immunity, potentially leading to immune escape (56). Tumor infiltrating lymphocytes (TIL) particularly demonstrate significant anti-tumor activity, with higher TIL densities associated with improved patient survival (57, 58). These complementary roles of platelet and lymphocytes underscore PLR’s potential as an integrated prognostic indicator, suggesting that patients with elevated PLR may benefit from more intensive therapy and surveillance protocols.

Certain limitations must be acknowledged when interpreting our results. First, the inclusion of only retrospective studies may introduce inherent biases in our analysis, and the relatively modest sample size precluded comprehensive subgroup analyses. Further prospective investigations are essential for validation. Second, studies currently employ diverse approaches to establish threshold values, introducing potential methodological inconsistencies influenced by demographic and clinical variables. Future research would benefit from developing either universal standards or specialized cutoff criteria that account for specific patient populations and clinical contexts. Third, the observed publication bias in RFS analyses, referring to the omission of studies with negative or inconclusive results due to their lower likelihood of publication, may have impact the reliability of our findings. This bias may be attributable to factors such as small sample sizes. Finally, the limited number of studies reporting certain survival outcomes (PFS, OS, and CSS) necessitates additional research to strengthen these findings.

## 5 Conclusion

This meta-analysis provides evidence that elevated PLR notably correlates with RFS and PFS in NMIBC patients. Given its economic efficiency and routine availability in clinical settings, PLR shows promise as a valuable marker for NMIBC patient care decisions. Nevertheless, robust prospective studies involving larger populations are essential to validate these initial observations.

## Data Availability

The original contributions presented in the study are included in the article/**Supplementary Material**. Further inquiries can be directed to the corresponding author.
