# Supplementary material for: Elevated platelet-to-lymphocyte ratio predicts poor clinical outcomes in non-muscle invasive bladder cancer: a systematic review and meta-analysis
Source: Front Immunol. 2025 May 13;16:1578069. doi: 10.3389/fimmu.2025.1578069 (PMC12106335; doi:10.3389/fimmu.2025.1578069)
Supplement: Supplementary file 1 [file Table1.docx]

**Supplementary Tables**

**Supplementary Table 1** The search strategy

**Pubmed**

| **Search number** | **Query** | **Results** |
| --- | --- | --- |
| 1 | Non-Muscle Invasive Bladder Neoplasms[MeSH Terms] | 356 |
| 2 | "bladder cancer without muscle invasion"[Title/Abstract] OR "NMIBC"[Title/Abstract] OR "non muscle invasive bladder cancer"[Title/Abstract] OR "non muscle invasive bladder carcinoma"[Title/Abstract] OR "Non Muscle Invasive Bladder Neoplasms"[Title/Abstract] OR "non muscle invasive bladder tumor"[Title/Abstract] OR "non muscle invasive bladder tumour"[Title/Abstract] OR "non muscle invasive urinary bladder cancer"[Title/Abstract] OR "non muscle invasive urinary bladder carcinoma"[Title/Abstract] OR "nonmuscle invasive bladder cancer"[Title/Abstract] OR "nonmuscle invasive bladder carcinoma"[Title/Abstract] OR "nonmuscle invasive cancer of the bladder"[Title/Abstract] | 4,852 |
| 3 | "platelet lymphocyte ratio"[Title/Abstract] OR "platelet to lymphocyte ratio"[Title/Abstract] OR "platelet/lymphocyte ratio"[Title/Abstract] OR "PLR lymphocyte "[Title/Abstract] OR "thrombocyte lymphocyte ratio"[Title/Abstract] OR "platelet lymphocyte ratio"[Title/Abstract] OR "PLR"[Title/Abstract] | 8,534 |
| 4 | (#1 OR #2) AND #3 | 24 |

**Embase**

| No. | Query | Results |
| --- | --- | --- |
| #5 | (#1 OR #2) AND (#3 OR #4) | 43 |
| #4 | 'platelet to lymphocyte ratio':ab,ti,kw OR 'platelet/lymphocyte ratio':ab,ti,kw OR 'plr lymphocyte':ab,ti,kw OR 'thrombocyte lymphocyte ratio':ab,ti,kw OR 'platelet lymphocyte ratio':ab,ti,kw OR 'plr':ab,ti,kw | 12130 |
| #3 | 'platelet lymphocyte ratio'/exp | 9751 |
| #2 | 'bladder cancer without muscle invasion':ab,ti,kw OR 'nmibc':ab,ti,kw OR 'non muscle invasive bladder cancer':ab,ti,kw OR 'non muscle invasive bladder carcinoma':ab,ti,kw OR 'non muscle invasive bladder neoplasms':ab,ti,kw OR 'non muscle invasive bladder tumor':ab,ti,kw OR 'non muscle invasive bladder tumour':ab,ti,kw OR 'non muscle invasive urinary bladder cancer':ab,ti,kw OR 'non muscle invasive urinary bladder carcinoma':ab,ti,kw OR 'nonmuscle invasive bladder cancer':ab,ti,kw OR 'nonmuscle invasive bladder carcinoma':ab,ti,kw OR 'nonmuscle invasive cancer of the bladder':ab,ti,kw | 8520 |
| #1 | 'non muscle invasive bladder cancer'/exp | 7377 |

**Cocharen**

| ID | Search | Hits |
| --- | --- | --- |
| #1 | MeSH descriptor: [Non-Muscle Invasive Bladder Neoplasms] explode all trees | 77 |
| #2 | ('bladder cancer without muscle invasion' OR 'NMIBC' OR 'non muscle invasive bladder cancer' OR 'non muscle invasive bladder carcinoma' OR 'Non Muscle Invasive Bladder Neoplasms' OR 'non muscle invasive bladder tumor' OR 'non muscle invasive bladder tumour' OR 'non muscle invasive urinary bladder cancer' OR 'non muscle invasive urinary bladder carcinoma' OR 'nonmuscle invasive bladder cancer' OR 'nonmuscle invasive bladder carcinoma' OR 'nonmuscle invasive cancer of the bladder'):ab,ti,kw | 1230 |
| #3 | ('platelet lymphocyte ratio' OR 'platelet to lymphocyte ratio' OR 'platelet lymphocyte ratio' OR 'PLR lymphocyte ' OR 'thrombocyte lymphocyte ratio' OR 'platelet lymphocyte ratio' OR 'PLR'):ab,ti,kw | 622 |
| #4 | (#1 OR #2) AND #3 | 2 |

**Web of science**

| # | Search |  | database |
| --- | --- | --- | --- |
| 1 | TS=((bladder cancer without muscle invasion) OR (NMIBC) OR (non muscle invasive bladder cancer) OR (non muscle invasive bladder carcinoma) OR (Non Muscle Invasive Bladder Neoplasms) OR (non muscle invasive bladder tumor) OR (non muscle invasive bladder tumour) OR (non muscle invasive urinary bladder cancer) OR (non muscle invasive urinary bladder carcinoma) OR (nonmuscle invasive bladder cancer) OR (nonmuscle invasive bladder carcinoma) OR (nonmuscle invasive cancer of the bladder)) | Web of Science | 8363 |
| 2 | TS=((platelet lymphocyte ratio) OR (platelet to lymphocyte ratio) OR (platelet/lymphocyte ratio) OR (PLR lymphocyte ) OR (thrombocyte lymphocyte ratio) OR (platelet lymphocyte ratio) OR (PLR)) | Web of Science | 14179 |
| 3 | #2 AND #1 | Web of Science | 38 |

**Supplementary Table 2** Reasons for exclusion of rescreened literature

| First author | Publication time | Study design | Region | Sample size | Type of patient | Exclusion grounds |
| --- | --- | --- | --- | --- | --- | --- |
| Mateusz Adamkiewicz(1) | 2021 | Observational study | Poland | 125 | patients with nonmuscle invasive bladder cancer who received BCG immunotherapy. | The HR value of PLR cannot be calculated according to the information in the article |
| Serkan Akan(2) | 2021 | Observational study | Turkey | 96 | High-risk NMIBC treated with BCG for at least 6 months | The HR value of PLR cannot be calculated according to the information in the article |
| Yavuz Tarik Atik(3) | 2021 | Observational study | Turkey | 221 | primary NMIBC with TURBT | The HR value of PLR cannot be calculated according to the information in the article |
| Osman Barut(4) | 2021 | Observational study | Turkey | 112 | patients who underwent transurethral resection for the diagnosis of bladder cancer | The population included patients with myoinvasive bladder cancer without PLR predictors |
| Deniz Bolat(5) | 2023 | Observational study | Turkey | 269 | patients with intermediate- and high-risk nonmuscle-invasive bladder cancer (NMIBC) | The HR value of PLR cannot be calculated according to the information in the article |
| Francesco Cantiello(6) | 2018 | Observational study | Italy | 1155 | patients with high-risk NMIBC | The HR value of PLR cannot be calculated according to the information in the article |
| Huaping Chen(7) | 2022 | Observational study | China | 208 | NMIBC patients who underwent TURBT and postoperative intravesical chemotherapy | The HR value of PLR cannot be calculated according to the information in the article |
| Francesco Chiancone(8) | 2021 | Observational study | Italy | 72 | patients with NMIBC with BCG failure or intolerance who underwent HIVEC at our institution | The HR value of PLR cannot be calculated according to the information in the article |
| Mehmet Kaynar(9) | 2014 | Observational study | Turkey | 291 | non-muscle-invasive bladder cancer (NMIBC) and muscleinvasive disease (MIBC) patients | The population included patients with myoinvasive bladder cancer, and the HR value of PLR could not be calculated based on the information in the article |
| Su-Min Lee(10) | 2015 | Observational study | UK | 226 | 175 and 51 with NMIBC and MIBC | The population included patients with myoinvasive bladder cancer, and the HR value of PLR could not be calculated based on the information in the article |
| Peng Liu(11) | 2022 | Observational study | China | 183 | intermediate and high risk NMIBC | The HR value of PLR cannot be calculated according to the information in the article |
| Ertuğrul Şefik(12) | 2018 | Observational study | Turkey | 112 | patients with organconffned invasive bladder tumors (T2) detected at histopathological examination of transuretral resection material | The population did not meet the inclusion criteria and the HR value of the PLR could not be calculated from the information in the article |
| Avnish Kumar Singh(13) | 2024 | Observational study | India | 69 | MIBC (n = 44) and NMIBC (n = 25) | The population did not meet the inclusion criteria and the HR value of the PLR could not be calculated from the information in the article |
| Xingxing Tang(14) | 2020 | Observational study | China | 641 | patients with Ta (57.57%) and T1 (25.43%) tumors, MIBC (15.91%) | The population did not meet the inclusion criteria and the HR value of the PLR could not be calculated from the information in the article |
| Hüseyin Alperen Yıldız(15) | 2021 | Observational study | Turkey | 94 | NMIBC | The HR value of PLR cannot be calculated according to the information in the article |
| Matteo Ferro(16) | 2021 | editorial | Italy | NA | NMIBC and MIBC | The population did not meet the inclusion criteria and the HR value of the PLR could not be calculated from the information in the article |
| Joseph Plasek(17) | 2021 | Observational study | United States | 1331 | patients (stage Ta, T1, or carcinoma in situ) who underwent TURBT | No PLR predictors were found |
| Kaushal Kumar Rai(18) | 2024 | Observational study | India | 100 | patients with NMIBC who were undergoing TURBT | The HR value of PLR cannot be calculated according to the information in the article |
| Yutong Song(19) | 2022 | Observational study | China | 371 | patients with NMIBC who were undergoing TURBT | No PLR predictors were found |
| A. S. Escandón(20) | 2019 | Observational study | / | 43 | patients with upper-intermediate risk NMIBC treated with neoadjuvant HIVEC | Full text not available |
| H. N. Fu(21) | 2021 | Observational study | China | 271 | NMIBC | Non-English articles |

1. Adamkiewicz M, Bryniarski P, Kowalik M, Burzynski B, Rajwa P, Paradysz A. Lymphocyte-to-Monocyte Ratio Is the Independent Prognostic Marker of Progression in Patients Undergoing BCG-Immunotherapy for Bladder Cancer. Frontiers in Oncology. 2021;11.

2. Akan S, Ediz C, Sahin A, Tavukcu HH, Urkmez A, Horasan A, et al. Can the systemic immune inflammation index be a predictor of BCG response in patients with high-risk non-muscle invasive bladder cancer? Int J Clin Pract. 2021;75(4):e13813.

3. Atik YT, Cimen HI, Gul D, Arslan S, Kose O, Halis F. Are the preoperative neutrophil/lymphocyte ratio and platelet/lymphocyte ratio predictive for lamina propria invasion in aging patients? Aging Male. 2020;23(5):1528-32.

4. Barut O, Resim S. Can immature granulocyte predict the prognosis of bladder cancer? Medical Science. 2021;25(109):723-9.

5. Bolat D, Baltaci S, Akgul M, Karabay E, Izol V, Aslan G, et al. Predictive Role of the Systemic Immune Inflammation Index for Intravesical BCG Response in Intermediate- and High-Risk Non-Muscle-Invasive Bladder Cancer. Urologia Internationalis. 2023;107(6):617-23.

6. Cantiello F, Russo GI, Vartolomei MD, Farhan ARA, Terracciano D, Musi G, et al. Systemic Inflammatory Markers and Oncologic Outcomes in Patients with High-risk Non-muscle-invasive Urothelial Bladder Cancer. Eur Urol Oncol. 2018;1(5):403-10.

7. Chen H, Wu X, Wen Z, Zhu Y, Liao L, Yang J. The Clinicopathological and Prognostic Value of NLR, PLR and MLR in Non-Muscular Invasive Bladder Cancer. Arch Esp Urol. 2022;75(5):467-71.

8. Chiancone F, Fabiano M, Carrino M, Fedelini M, Meccariello C, Fedelini P. Impact of systemic inflammatory markers on the response to Hyperthermic IntraVEsical Chemotherapy (HIVEC) in patients with non-muscle-invasive bladder cancer after bacillus Calmette-Guérin failure. Arab J Urol. 2021;19(1):86-91.

9. Kaynar M, Yıldırım ME, Badem H, Caviş M, Tekinarslan E, Istanbulluoğlu MO, et al. Bladder cancer invasion predictability based on preoperative neutrophil-lymphocyte ratio. Tumour Biol. 2014;35(7):6601-5.

10. Lee SM, Russell A, Hellawell G. Predictive value of pretreatment inflammation-based prognostic scores (neutrophil-to-lymphocyte ratio, platelet-to-lymphocyte ratio, and lymphocyte-to-monocyte ratio) for invasive bladder carcinoma. Korean J Urol. 2015;56(11):749-55.

11. Liu P, Chen S, Gao X, Liang H, Sun D, Shi B, et al. Preoperative sarcopenia and systemic immune-inflammation index can predict response to intravesical Bacillus Calmette-Guerin instillation in patients with non-muscle invasive bladder cancer. Front Immunol. 2022;13:1032907.

12. Şefik E, Günlüsoy B, Aydoğdu Ö, Topçu YK, Ceylan Y, Değirmenci T, et al. Predictive role of neutrophil-to-lymphocyte ratio on upstaging of organ-confined invasive urothelial bladder cancer to non-organ-confined disease. Turk J Urol. 2018;44(2):119-24.

13. Singh AK, Sarma D, Phukan M, Bagchi PK, Barua SK. Correlation of Serum Lymphocyte-Derived Biomarkers in Muscle Invasive and Non-Muscle Invasive Bladder Cancer: a Hospital Based Retrospective Study. Indian Journal of Surgery. 2024;86(3):587-91.

14. Tang X, Cao Y, Liu J, Wang S, Yang Y, Du P. Diagnostic Value of Inflammatory Factors in Pathology of Bladder Cancer Patients. Front Mol Biosci. 2020;7:575483.

15. Yıldız HA, Değer MD, Aslan G. Prognostic value of preoperative inflammation markers in non-muscle invasive bladder cancer. Int J Clin Pract. 2021;75(6):e14118.

16. Ferro M, Caputo VF, Barone B, Imbimbo C, de Cobelli O, Crocetto F. Lymphocyte to Monocyte Ratio: A New Independent Prognostic Factor in Bladder Cancer Progression? Front Oncol. 2021;11:754649.

17. Plasek J, Weissert J, Downs T, Richards K, Ravvaz K. Clinicopathological Criteria Predictive of Recurrence Following Bacillus Calmette-Guérin Therapy Initiation in Non-Muscle-Invasive Bladder Cancer: Retrospective Cohort Study. JMIR Cancer. 2021;7(2):e25800.

18. Rai KK, Tyagi VK, Tyagi S. Predictive Factors for Early Recurrence after Transurethral Resection in Non-Muscle Invasive Bladder Cancer. International Journal of Pharmaceutical and Clinical Research. 2024;16(6):1986-90.

19. Song Y, Tian J, Yang L, Zhang Y, Dong Z, Ding H, et al. Prognostic value of preoperative platelet-related parameters and plasma fibrinogen in patients with non-muscle invasive bladder cancer after transurethral resection of bladder tumor. Future Oncol. 2022;18(26):2933-42.

20. Escandón AS, Mata JL, Sousa-González D, Gómez SR, Díaz IP. Recirculating endovesical chemohyperthermia in the treatment of non-muscle invasive bladder cancer. Medicina Balear. 2019;34(2):33-9.

21. Fu HN, Liang PY, Wang SX, Fu JS. Construction of a marker scoring system for predicting the recurrence of non-muscular invasive urothelial carcinoma of the bladder. Chinese Journal of Cancer Prevention and Treatment. 2021;28(3):230-6.

**Supplementary Table** **3** Results of NOS scale

**NEWCASTLE - OTTAWA QUALITY ASSESSMENT SCALE**

**COHORT STUDIES**

Note: A study can be awarded a maximum of one star for each numbered item within the Selection and Outcome categories. A maximum of two stars can be given for Comparability

**Selection**

1) Representativeness of the exposed cohort

a) truly representative of the average _______________ (describe) in the community ****

b) somewhat representative of the average ______________ in the community ****

c) selected group of users eg nurses, volunteers

d) no description of the derivation of the cohort

2) Selection of the non exposed cohort

a) drawn from the same community as the exposed cohort ****

b) drawn from a different source

c) no description of the derivation of the non exposed cohort

3) Ascertainment of exposure

a) secure record (eg surgical records) ****

b) structured interview ****

c) written self report

d) no description

4) Demonstration that outcome of interest was not present at start of study

a) yes ****

b) no

**Comparability**

1) Comparability of cohorts on the basis of the design or analysis

a) study controls for _____________ (select the most important factor) ****

b) study controls for any additional factor **** (This criteria could be modified to indicate specific control for a second important factor.)

**Outcome**

1) Assessment of outcome

a) independent blind assessment ****

b) record linkage ****

c) self report

d) no description

2) Was follow-up long enough for outcomes to occur

a) yes (select an adequate follow up period for outcome of interest) ****

b) no

3) Adequacy of follow up of cohorts

a) complete follow up - all subjects accounted for ****

b) subjects lost to follow up unlikely to introduce bias - small number lost - > ____ % (select an adequate %) follow up, or description provided of those lost) ****

c) follow up rate < ____% (select an adequate %) and no description of those lost

d) no statement

NOS scale scores of 11 studies included in this meta-analysis

| Author, year | selection | | | | Comparability | Outcome | | | Total Score |
| --- | --- | --- | --- | --- | --- | --- | --- | --- | --- |
|  | 1) | 2) | 3) | 4) | 1) | 1) | 2) | 3) |  |
| A. Çaglayan 2023 | 1 | 1 | 1 | 0 | 2 | 1 | 1 | 1 | 8 |
| M. A. Chakra 2024 | 1 | 1 | 1 | 0 | 1 | 1 | 1 | 1 | 7 |
| L. Ding 2023 | 1 | 1 | 1 | 0 | 1 | 1 | 1 | 1 | 7 |
| M. Kang 2017 | 1 | 1 | 1 | 0 | 2 | 1 | 1 | 1 | 8 |
| S. Y. Mao 2017 | 1 | 1 | 1 | 0 | 2 | 1 | 1 | 1 | 8 |
| C. Wang 2023 | 1 | 1 | 1 | 0 | 2 | 1 | 1 | 1 | 8 |
| X. Y. Wang 2024 | 1 | 1 | 1 | 0 | 2 | 1 | 1 | 1 | 8 |
| R. Wu 2022 | 1 | 1 | 1 | 0 | 2 | 1 | 1 | 1 | 8 |
| X. Yi 2023 | 1 | 1 | 1 | 0 | 2 | 1 | 1 | 1 | 8 |
| I. Ö. Yilmaz 2024 | 1 | 1 | 1 | 0 | 2 | 1 | 1 | 1 | 8 |
| H. D. Yuk 2019 | 1 | 1 | 1 | 0 | 2 | 1 | 1 | 1 | 8 |
